# SGK1 negatively regulates inflammatory immune responses and protects against alveolar bone loss through modulation of TRAF3 activity

**DOI:** 10.1016/j.jbc.2022.102036

**Published:** 2022-05-17

**Authors:** Xiao Han, Junling Ren, Hannah Lohner, Lan Yakoumatos, Ruqiang Liang, Huizhi Wang

**Affiliations:** 1 Department of Oral and Craniofacial Molecular Biology, VCU Philips Institute for Oral Health Research, Virginia Commonwealth University, Richmond, Virginia, USA; 2 Department of Oral Immunology and Infectious Diseases, University of Louisville, Louisville, Kentucky, USA; 3 Department of Biochemistry and Molecular Medicine, University of California, Davis, Davis, California, USA

**Keywords:** SGK1, inflammation, periodontal bone loss, TRAF3, ubiquitin E3 ligase, BMDM, bone marrow–derived macrophage, cIAP1, cellular inhibitor of apoptosis protein 1, CO_2_, carbon dioxide, GSK3β, glycogen synthase kinase 3 beta, HECT, homologous to E6AP C terminus, IC, intercellular, IL, interleukin, MIP-2, macrophage inflammatory protein 2, mTORC, mammalian target of rapamycin complex, NDRG1, N-myc downstream–regulated gene 1, Nedd4, neural precursor cell–expressed developmentally downregulated 4, NIK, NF-κB–inducing kinase, qPCR, quantitative PCR, SGK1, serum- and glucocorticoid-regulated kinase 1, Smurf1, Smad ubiquitin regulatory factor 1, TLR, Toll-like receptor, TNFAIP3, tumor necrosis factor α–induced protein 3, TRAF, tumor necrosis factor receptor–associated factor, VCU, Virginia Commonwealth University

## Abstract

Serum- and glucocorticoid-regulated kinase 1 (SGK1) is a serine/threonine kinase that plays important roles in the cellular stress response. While SGK1 has been reported to restrain inflammatory immune responses, the molecular mechanisms involved remain elusive, especially in oral bacteria-induced inflammatory milieu. Here, we found that SGK1 curtails *Porphyromonas gingivalis*–induced inflammatory responses through maintaining levels of tumor necrosis factor receptor-associated factor (TRAF) 3, thereby suppressing NF-κB signaling. Specifically, SGK1 inhibition significantly enhances production of proinflammatory cytokines, including tumor necrosis factor α, interleukin (IL)-6, IL-1β, and IL-8 in *P. gingivalis*–stimulated innate immune cells. The results were confirmed with siRNA and LysM-Cre–mediated *SGK1* KO mice. Moreover, *SGK1* deletion robustly increased NF-κB activity and c-Jun expression but failed to alter the activation of mitogen-activated protein kinase signaling pathways. Further mechanistic data revealed that *SGK1* deletion elevates TRAF2 phosphorylation, leading to TRAF3 degradation in a proteasome-dependent manner. Importantly, siRNA-mediated *traf3* silencing or c-Jun overexpression mimics the effect of SGK1 inhibition on *P. gingivalis*–induced inflammatory cytokines and NF-κB activation. In addition, using a *P. gingivalis* infection–induced periodontal bone loss model, we found that SGK1 inhibition modulates TRAF3 and c-Jun expression, aggravates inflammatory responses in gingival tissues, and exacerbates alveolar bone loss. Altogether, we demonstrated for the first time that SGK1 acts as a rheostat to limit *P. gingivalis*–induced inflammatory immune responses and mapped out a novel SGK1–TRAF2/3–c-Jun–NF-κB signaling axis. These findings provide novel insights into the anti-inflammatory molecular mechanisms of SGK1 and suggest novel interventional targets to inflammatory diseases relevant beyond the oral cavity.

Homeostasis between proinflammatory and anti-inflammatory response is critical to the outcome of immune responses and progression of many inflammatory diseases. Concomitant to initiation of proinflammatory responses, anti-inflammatory mechanisms are also ignited to restrain the overwhelming inflammation (1, 2). In this regard, our and other studies have demonstrated that activation of a variety of signaling molecules, such as PI3K, PKB/Akt, mammalian target of rapamycin complex (mTORC) 1, tumor necrosis factor α–induced protein 3 (TNFAIP3/A20), glycogen synthase kinase 3 beta (GSK3β), Janus kinase 3, and wingless-related integration site 3a, restrains the magnitude and intensity of inflammation (3, 4, 5, 6, 7). With advances in understanding the mechanism of anti-inflammatory machinery, it is becoming evident that anti-inflammatory response is almost always activated concurrently with proinflammatory response, lasts from several seconds to several years, and acts as a positive process rather than a negative one (1, 2).

Periodontitis is a chronic inflammatory disease characterized by severe gingival inflammation and destruction of alveolar bone and connective tissues surrounding the teeth. It is estimated that over half of the adult population in the United States has periodontitis (8). In oral cavity, the balance of proinflammatory and anti-inflammatory responses ensures a state of immune homeostasis, which is critical for the maintenance of gingival health. Tissue destruction is primarily a consequence of excessive intensity and/or prolonged duration of host immune inflammatory responses to dysbiotic microbiota. It was reported that the increased proinflammatory cytokines, including TNFα, IL-12, and IL-1β (9, 10, 11), and decreased anti-inflammatory cytokines, including IL-10, IL-4, and IL-1Ra, are relevant to the periodontal health (12). *Porphyromonas gingivalis* is considered as a typical pathogenic bacterium, and its colonization in the oral community induces dysregulation of the inflammatory immune response and, ultimately causing irreversible damage to the periodontal tissues (13, 14, 15). Apart from periodontal inflammation, *P. gingivalis* infection was also reported to increase the risk of several serious systemic diseases, such as atherosclerosis, diabetes mellitus, rheumatoid arthritis, and oral cancer (16, 17), highlighting the importance of elucidating regulatory mechanisms of *P. gingivalis*–mediated inflammation. While *P. gingivalis* was found to induce proinflammatory responses through binding to pattern recognition receptors on host immune cells, the *P. gingivalis*–mediated anti-inflammatory immune mechanisms and their functional role in the overall periodontal disease process remain less explored.

*P. gingivalis* frequently exploits host machinery, especially the amount and/or activity of protein kinases, such as β-catenin, mTORC1, or Akt, and by which it can modulate the course and magnitude of inflammatory responses (7, 18, 19). Serum- and glucocorticoid-regulated kinase 1 (SGK1) is a ubiquitous serine/threonine kinase that was originally considered to function in regulation of sodium homeostasis. Recent studies found that SGK1 was associated to many other diseases, such as hypertension, diabetes, recurrent pregnancy loss, multiple sclerosis, and periodontitis (20, 21, 22, 23, 24). Activation of SGK1 is through PI3K pathway with being phosphorylated by phosphoinositide-dependent kinase 1 at threonine 256 within the kinase domain and mTORC2 at serine 422 within the C-terminal hydrophobic motif and thereby engages in regulation of innate and adaptive immune responses (23, 25, 26, 27, 28). Our and other recent studies have demonstrated that SGK1 negatively regulates Toll-like receptor (TLR)–mediated inflammatory responses and acts as a key regulator in macrophage polarization (22, 27, 29). However, the role of SGK1 in *P. gingivalis*–induced inflammation and the underlying molecular mechanisms remain largely unknown.

Tumor necrosis factor receptor–associated factors (TRAFs) are a large family of intracellular (IC) signaling molecules that include six typical members (TRAF1–6) and one atypical member (TRAF7) in mammalian cells (30, 31). Originally identified as signaling adaptors, the TRAF molecules are now known to act as ubiquitin E3 ligases (E3s) and mediate a large variety of signaling transduction upon activation of immune receptors, such as pattern-recognition receptors, antigen receptors, cytokine receptors and, importantly, TNF receptor superfamily members (32, 33). All TRAF members but TRAF1 have been revealed to contain a RING domain located in the N-terminal region. This is key to mediate protein ubiquitination as well as oligomerization and association of TRAF members with their upstream receptors, adaptors, and downstream effector proteins. Through the lysine (K)-48 and K63-linked polyubiquitin chain, TRAF members control multiple signaling pathways that in turn are important for the induction of genes associated with innate immunity, inflammation, and cell survival (34, 35). Recent studies demonstrated that some TRAF proteins, especially TRAF2 and TRAF3, could function as negative regulators in NF-κB signaling pathways, in which their E3 activity is key for their regulatory roles (35, 36, 37). On the other hand, our and other studies found that some protein serine and threonine kinases such as SGK1 and Janus kinase 3 regulate the activity of an ubiquitin E3 ligase, Nedd4 (neural precursor cell–expressed developmentally downregulated 4)-2, and thereby control the activity of downstream inflammatory signaling pathways (5, 38, 39). Moreover, *P. gingivalis* infection was closely associated with activation of SGK1 and multiple E3 ligases such as Nedd4-2 and Smurf1 (Smad ubiquitin regulatory factor 1) (5, 40). Surprisingly, there are no studies about the possible activation of TRAF2/3 in *P. gingivalis*–induced inflammatory responses nor about whether SGK1 affects E3 activity of TRAF members, and thereby functions in the regulation of inflammation.

In this study, we demonstrated for the first time that SGK1 acts as a gatekeeper to restrain the inflammatory immune response in cultured cells and a *P. gingivalis*–induced periodontal bone loss model. Moreover, our mechanistic data identified that SGK1 inhibition enhances expression of c-Jun and phosphorylation of TRAF2, which in turn elevates the degradation of TRAF3, diminishes its suppressive effect on NF-κB, and ultimately aggravates *P. gingivalis*–mediated inflammatory immune responses. In addition, SGK1 inhibition aggravates *P. gingivalis*–induced periodontal inflammation in mice gingiva and exacerbates subsequent alveolar bone loss, suggesting that the anti-inflammatory signaling axis, SGK1–TRAF2/3–c-Jun/NF-κB, could be targeted for the development of novel interventional therapeutics to control periodontitis and other inflammatory diseases beyond the oral cavity.

## Results

### Infection with *P. gingivalis* leads to activation of SGK1 in innate immune cells

**Figure 1:**
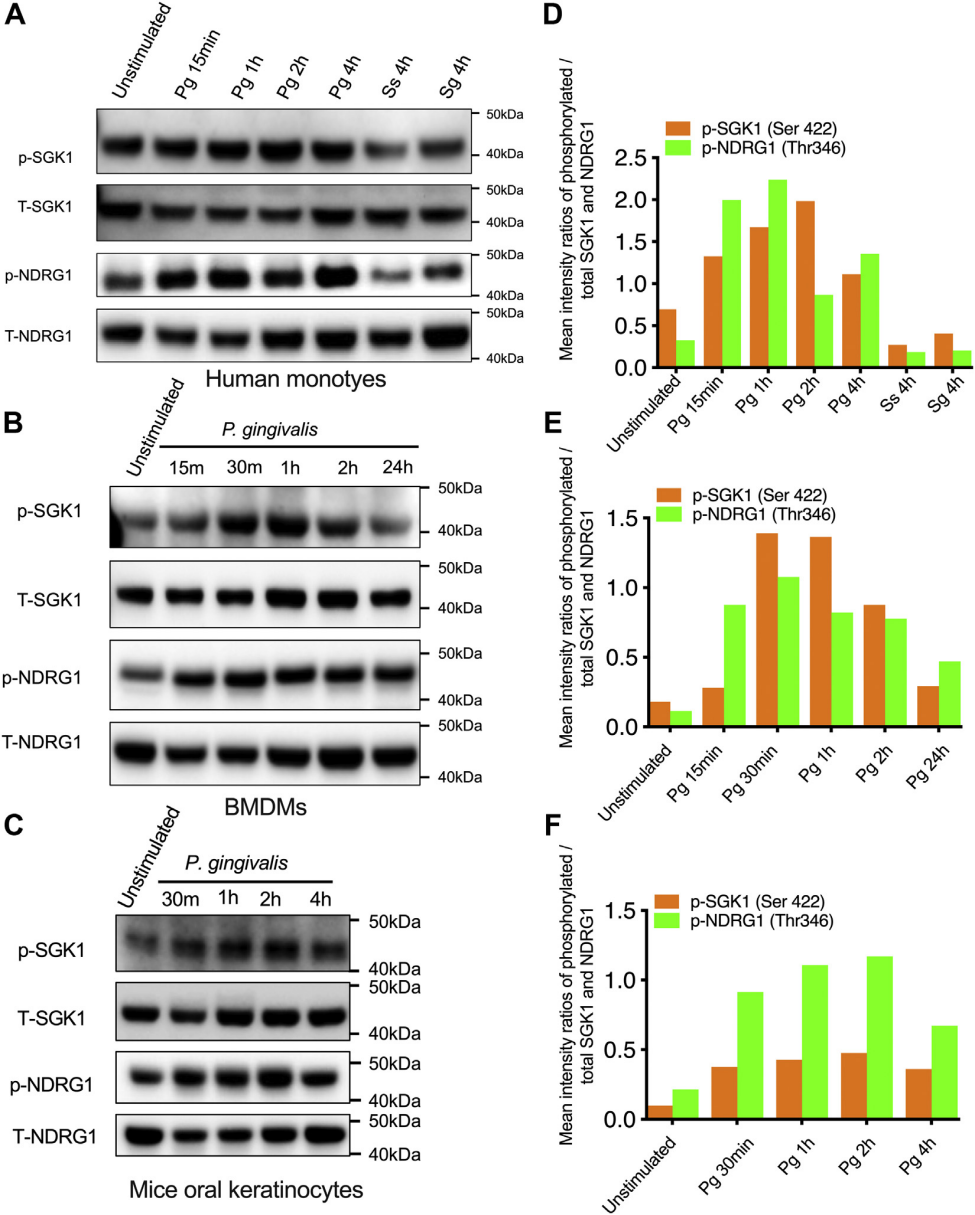
**Infection with *Porphyromonas gingivalis* leads to activation of SGK1 in innate immune cells.** Primary human monocytes (*A*), BMDMs (*B*), and primary mice keratinocytes (*C*) were stimulated with *P. gingivalis* 33277 (Pg), *Streptococcus gordonii* DL1 (Sg), or *Streptococcus sanguinis* (Ss) at MOI of 10, for the time indicated. The whole cell lysates were collected for Western blotting, and the immunoblots were probed for phosphorylation and total expression of SGK1 and its prototypical substrate, NDRG1, in human monocytes (*A*), BMDMs (*B*), and primary keratinocytes (*C*), respectively. *D*–*F*, the intensity ratios of phosphorylation of SGK1 or NDRG1 relative to total SGK1 or NDRG1 for human monocytes (*D*), BMDMs (*E*), and primary keratinocytes (*F*) were determined by densitometry quantification assay. All the blots shown are representative of 3 to 5 independent experiments. BMDM, bone marrow–derived macrophage; MOI, multiplicity of infection; NDRG1, N-myc downstream–regulated gene 1; SGK1, serum- and glucocorticoid-regulated kinase 1.

**Supplemental Figure S1:**
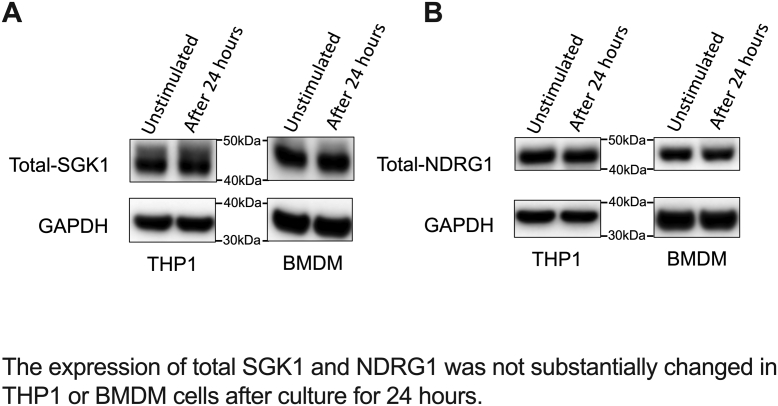


Our previous studies have demonstrated that SGK1 promotes macrophage polarization to M2 and restrains secretion of TLR-mediated inflammatory cytokines (22, 27, 29). *P. gingivalis* is a key pathogenic bacterium that triggers periodontitis and was associated with multiple inflammatory diseases, such as arthritis, atherosclerosis, and Alzheimer’s disease (41, 42, 43). To examine if SGK1 also functions in *P. gingivalis*–induced inflammation and elucidate the molecular mechanisms involved, we first examined activation of SGK1 in response to the challenge of *P. gingivalis* in different innate cells. We found that *P. gingivalis* infection enhanced phosphorylation of SGK1 at serine 422 in human monocytes (Fig. 1, *A* and *D*), bone marrow–derived macrophages (BMDMs) (Fig. 1, *B* and *E*), and primary oral epithelial cells (Fig. 1, *C* and *F*) throughout all the time points measured (up to 24 h). In contrast, other oral bacteria including *Streptococcus gordonii* and *Streptococcus sanguinis* failed to do so (Fig. 1, *A* and *D*). To further confirm if *P. gingivalis* activated SGK1, we next examined phosphorylation of N-myc downstream regulated gene 1 (NDRG1), a *bona fide* substrate of SGK1 (44, 45). Like the phosphorylation of SGK1, infection of *P. gingivalis* indeed elevated phosphorylation of NDRG1 at threonine 346/356/366 in all cells we investigated (Fig. 1). Moreover, the expression of total SGK1 and NDRG1 was not substantially changed in human monocytes or BMDM cells (Fig. S1) after culture for 24 h, which substantiated the effect of SGK1 inhibition on the phosphorylation of SGK1 and NDRG1. Taken together, these results demonstrated that infection with *P. gingivalis* activates SGK1 in innate cells.

### SGK1 restrains *P. gingivalis*–induced inflammatory immune responses

**Figure 2:**
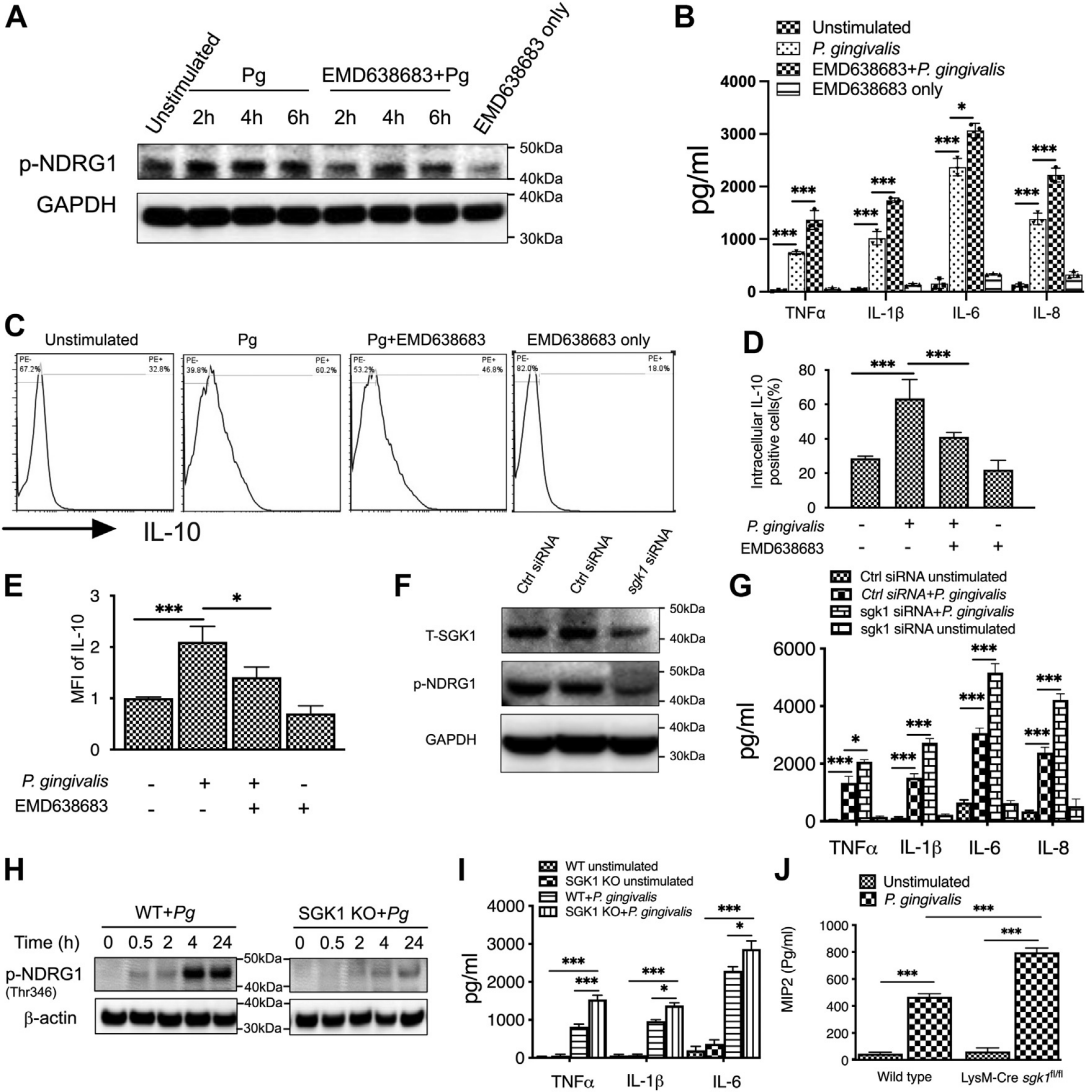
**Inhibition of SGK1 enhances *Porphyromonas gingivalis*–induced proinflammatory cytokines and reduces IL-10.** Human monocytes were pretreated with SGK1 inhibitor, EMD638683 (15 μM), for 2 h (*A*–*E*), or siRNA for 48 h (*F*) and then stimulated with *P. gingivalis* for 12 h followed by examination of the production of TNFα, IL-1β, IL-6, and IL-8 with ELISA and IL-10 with intracellular staining flow cytometry. *A*, Western blots showing the suppressive effect of EMD638683 on activity of SGK1 in *P. gingivalis*–stimulated human monocytes. *B*, ELISA showing the production of TNFα, IL-1β, IL-6, and IL-8 in the supernatants of *P. gingivalis*–stimulated human monocytes. *C*, typical fluorescence plots of flow cytometry showing intracellular staining cells with phycoerythrin-conjugated IL-10. *D* and *E*, percentage of cells expressing IL-10 (*D*) and the mean fluorescence intensity (MFI) that was normalized to the unstimulated group (*E*). *F*, Western blots showing the efficiency of siRNA-mediated *sgk1* gene silencing and phosphorylation of NDRG1. *G*, ELISA showing the production of TNFα, IL-1β, IL-6, and IL-8 in the supernatants from *P. gingivalis*–stimulated human monocytes with siRNA-mediated *sgk1* gene silencing. *H*, the efficacy of SGK1 deletion in BMDMs from wildtype or *LysM*-Cre *sgk1*^*fl/fl*^ mice. *I* and *J*, BMDMs generated from *LysM*-Cre *sgk1*^*fl/fl*^ or littermate control mice were stimulated with *P. gingivalis* for 12 h, then the production of proinflammatory cytokines including TNFα, IL-1β, IL-6 (*I*), and macrophage inflammatory protein 2 (MIP-2) (*J*) was measured with ELISA. All data represent the mean ± SEM of three independent experiments in duplicate. ∗ and ∗∗∗ represent *p* < 0.05 and *p* < 0.001, respectively. BMDM, bone marrow–derived macrophage; IL, interleukin; NDRG1, N-myc downstream–regulated gene 1; SGK1, serum- and glucocorticoid-regulated kinase 1; TNFα, tumor necrosis factor α.

Since *P. gingivalis* infection activates SGK1 in innate immune cells, we next examined if SGK1 affects production of inflammatory cytokines in response to *P. gingivalis* challenge. Using EMD638683, a specific SGK1 inhibitor, we found that pretreatment with SGK1 inhibitor drastically decreased phosphorylation of NDRG1 in response to the challenge of *P. gingivalis*, indicating the inhibitory efficacy of EMD638683 on SGK1 (Fig. 2*A*). Moreover, inhibition of SGK1 with EMD638683 significantly increased the secretion of TNFα, IL-6, IL-1β, and IL-8 in human monocytes upon the challenge with *P. gingivalis* (Fig. 2*B*). We also examined the possible effect of SGK1 on the secretion of IL-10, a prototypical anti-inflammatory cytokine, in *P. gingivalis*–stimulated monocytes. By using IC IL-10 staining and analysis with flow cytometry, we found that SGK1 inhibition significantly decreased IL-10 in *P. gingivalis*–stimulated human monocytes, represented by a decrease in positive cells and mean fluorescence intensity (Fig. 2, *C*–*E*), suggesting SGK1 may differentially regulate proinflammatory and anti-inflammatory cytokines. To exclude the possible unspecific effects of chemical inhibitor, we next utilized specific *sgk1* siRNA to verify the effects of SGK1 on the secretion of inflammatory cytokines. We found that silencing of SGK1 (Fig. 2*F*) significantly elevated the levels of TNFα, IL-6, IL-1β, and IL-8 production in *P. gingivalis*–stimulated human monocytes (Fig. 2*G*). Given the possible off-target effects of siRNA, we next utilized BMDMs from *LysM-Cre*^*+*^*sgk1*^*fl/fl*^ mice, in which the expression of SGK1 was efficiently expunged (Fig. 2*H*), represented by the disappearance of p-NDRG1, to confirm the influences of SGK1 on *P. gingivalis*–induced inflammatory cytokines. We found that SGK1 deficiency significantly elevated the secretion of proinflammatory cytokines, including TNFα, IL-1β, IL-6 (Fig. 2*I*), and macrophage inflammatory protein 2 (MIP-2) (Fig. 2*J*) in *P. gingivalis*–stimulated cells. Taken together, these results demonstrated that inhibition of SGK1 promotes production of *P. gingivalis*–induced proinflammatory cytokines while concurrently decreasing IL-10 secretion in innate immune cells, suggesting SGK1 may act as a rheostat to restrain *P. gingivalis*–mediated inflammatory immune responses.

### Inhibition of SGK1 promotes activity of NF-κB and increases expression of c-Jun in *P. gingivalis*–stimulated immune cells

**Figure 3:**
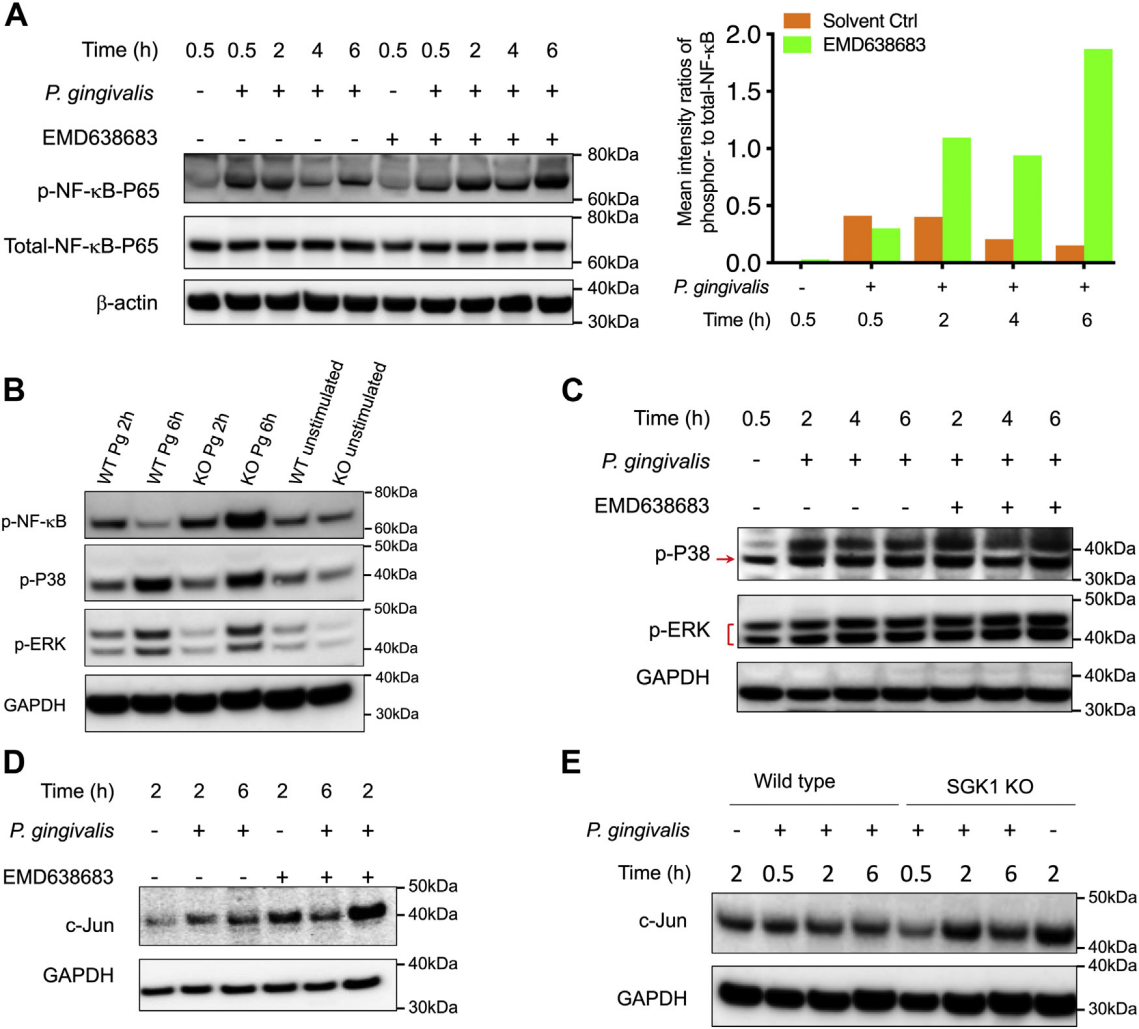
**SGK1 inhibition promotes *Porphyromonas gingivalis*–mediated NF-κB activity and c-Jun expression.** Human monocytes, pretreated with/without EMD638683 for 2 h (*A*, *C*, and *D*), and BMDMs (*B* and *E*) that were generated from *LysM-cre-sgk1*^*fl/fl*^ or littermate control mice were stimulated with *P. gingivalis* for the time indicated. Whole cell lysates were harvested for Western blotting assay, and the immunoblots were probed with antibodies to p-NF-κB, p-P38, p-ERK, and total c-Jun. The intensity ratio of phosphorylation of NF-κB to total NF-κB was determined by densitometry quantification assay. Antibodies to GAPDH were used as a loading control. *A*–*C*, Western blots showing SGK1 inhibition or deficiency enhances *P. gingivalis*–induced NF-κB activation (*A*) with densitometry quantification result (*A*, *right panel*) but does not affect p-P38 and p-ERK in monocytes (*C*) and BMDMs (*B*). *D* and *E*, inhibition or SGK1 deficiency leads to a remarkable increase of c-Jun in *P. gingivalis*–stimulated monocytes (*D*) and BMDMs (*E*) (this is the same blot as that for Fig. 4*A*). All the blots shown are representative of 3 to 5 independent experiments. BMDM, bone marrow–derived macrophage; ERK, extracellular-regulated kinase; SGK1, serum- and glucocorticoid-regulated kinase 1.

Since we have demonstrated that SGK1 restrains *P. gingivalis*–mediated inflammatory responses, we next sought to delineate how SGK1 affects inflammatory responses in innate immune cells upon the challenge of *P. gingivalis*. As shown in Figure 3*A*, we found that SGK1 inhibition robustly promotes the activity of NF-κB, represented by the phosphorylation of NF-κB P65 at serine 536, in *P. gingivalis*–stimulated monocytes. Notably, SGK1 inhibition–mediated increases of p-NF-κB were more evident for the samples with prolonged treatment (Fig. 3*A*, *right panel*). The incapability of the SGK1 inhibitor at early stage of treatments like 30 min might result from the proteolytic effect of gingipain, which could directly cleave NF-κB P65 and thus decrease its phosphorylation level at the early treated period. The results were consolidated by SGK1-deficient BMDMs (Fig. 3*B*). However, we did not observe the substantial changes of other prototypical inflammatory signaling pathways including phosphorylation of P38 and extracellular signal–regulated kinases in monocytes pretreated with EMD638683 (Fig. 3*C*) or SGK1-deficient BMDMs (Fig. 3*B*). In addition, SGK1 inhibition or *sgk1* gene deficiency drastically increased expression of c-Jun in *P. gingivalis*–stimulated human monocytes and BMDMs (Fig. 3, *D* and *E*). Given both NF-κB and c-Jun are key transcriptional factors that bind to the promoters of inflammatory cytokines, these results suggest that SGK1 inhibition–enhanced proinflammatory cytokine production in *P. gingivalis*–stimulated cells could arise from the increased NF-κB activity and c-Jun expression.

### SGK1 deficiency upregulates phosphorylation of TRAF2, reduces expression of TRAF3, and thereby promotes NF-κB activity in a ubiquitin-dependent manner

**Figure 4:**
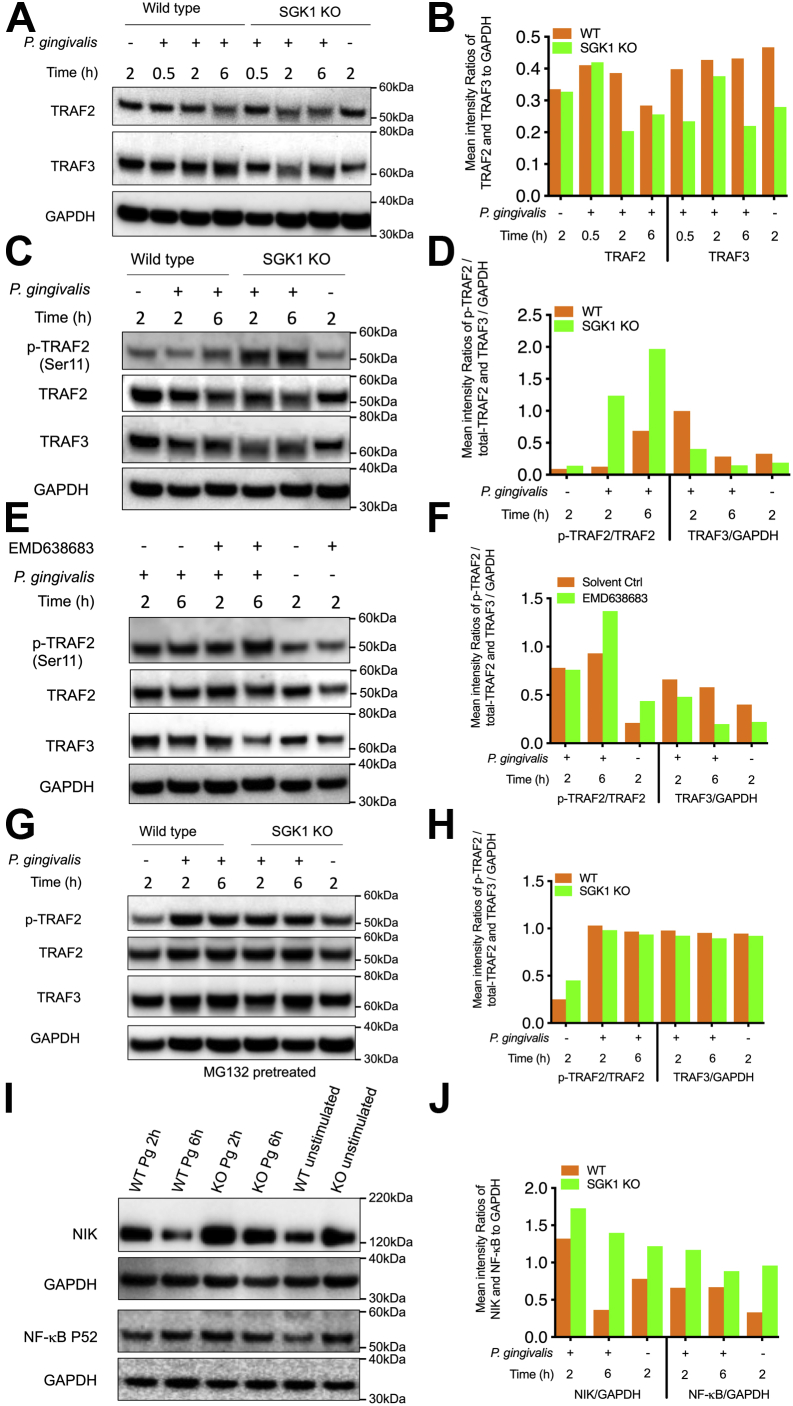
**Inhibition of SGK1 promotes that NF-κB activity is through enhancing phosphorylation of TRAF2 and subsequently reducing TRAF2 and TRAF3.** BMDMs generated from LysM-Cre *sgk1*^*fl/fl*^ or littermate control mice (*A*–*D* and *G*–*J*) or human monocytes (*E* and *F*) were stimulated with *Porphyromonas gingivalis* with/without pretreatment of EMD638683 (15 μM) for the time indicated. Whole-cell lysates were collected and analyzed by Western blotting. The intensity ratios of TRAF2, TRAF3, NIK, NF-κB P52, and phosphorylation of TRAF2 relative to GAPDH or total TRAF2 were determined by densitometry quantification assay. Representative immunoblots and densitometry results showing SGK1 deficiency (*A* and *B*) lead to a substantial decrease of TRAF2 and TRAF3, especially at 2 h postinfection (this is the same blot as that for Fig. 3*E*). *C* and *D*, enhances phosphorylation of TRAF2 at serine 11 in response to *P. gingivalis* challenge. *E* and *F*, Western blots showing that EMD638683 (15 μM) increases phosphorylation of TRAF2 and decreases TRAF3 in *P. gingivalis*–stimulated monocytes. The BMDMs above were also pretreated with MG-132 (10 μM) for 4 h and then stimulated with *P. gingivalis* followed by examination phosphor- and total TRAF2 and TRAF3 by Western blots. *G* and *H*, representative immunoblots showing that MG-132 abrogates the ability of SGK1 deficiency to enhance phosphorylation of TRAF2 and reduce TRAF2/3 in response to *P. gingivalis* challenge. *I* and *J*, Western blots showing the expression of NIK and NF-κB P52 in *P. gingivalis*–stimulated BMDMs from wildtype or SGK1 KO mice. The expression of GAPDH was used as a loading control for aforementioned analysis. All the blots shown are representative of 3 to 5 independent experiments. BMDM, bone marrow–derived macrophage; NIK, NF-κB–inducing kinase; SGK1, serum- and glucocorticoid-regulated kinase 1; TRAF, tumor necrosis factor receptor–associated factor.

Next we tested whether and how *P. gingivalis*–activated SGK1 affects TRAF2/3 in immune cells. As shown in Figure 4, *A* and *B*, SGK1 deficiency led to a drastic reduction of TRAF2 and TRAF3 in *P. gingivalis*–stimulated BMDMs, especially at 2 h postinfection. Since phosphorylation of TRAF2 is essential for degradation of TRAF3 and subsequently increased activity of NF-κB signaling (46), we next examined the effect of SGK1 activation on phosphorylation of TRAF2. As expected, SGK1 deficiency robustly increased phosphorylation of TRAF2 and substantially decreased expression of total TRAF2 and TRAF3 in *P. gingivalis*–stimulated BMDMs (Fig. 4, *C* and *D*). A similar trend was also observed in human monocytes with SGK1 inhibitor treatment (Fig. 4, *E* and *F*). To further determine if the alteration of TRAF2/3 was dependent on ubiquitin-mediated degradation, we utilized MG-132, a ubiquitination-associated proteasome inhibitor, to treat wildtype and SGK1-deficient BMDMs. We observed that MG-132 pretreatment abrogated the ability of SGK1 deficiency to increase TRAF2 phosphorylation and decrease TRAF2/3 expression in response to the challenge of *P. gingivalis*, indicating SGK1 protects TRAF3 against degradation, possibly through controlling its ubiquitination (Fig. 4, *G* and *H*). Interestingly, our results showed that SGK1 deficiency remarkably increases TRAF2 phosphorylation but decreases its total levels simultaneously, indicating phosphorylation of TRAF2 may lead to its own degradation upon challenge with *P. gingivalis*. Moreover, previous studies have also reported that TRAF2 deficiency aggravates inflammatory immune responses under some contexts (33, 36, 47), suggesting both TRAF2 and TRAF3 might be involved in regulation of *P. gingivalis*–induced inflammation. In addition, we found that SGK1 deficiency led to the accumulation of NF-κB–inducing kinase (NIK) and activation of NF-κB P52 in response to the challenge of *P. gingivalis* (Fig. 4, *I* and *J*), which suggests TRAF3 targets NIK for its constant degradation by the proteasome. Given SGK1 was reported to affect multiple ubiquitination E3 ligases such as Nedd4-2 and Smurf1 in other contexts (48, 49), it is possible that SGK1 may affect phosphorylation of TRAF2 and cIAP1 (cellular inhibitor of apoptosis protein 1) and subsequently impact TRAF3 (35, 50, 51). However, we did not observe substantial changes in cIAP1 in SGK1-deficient BMDMs (data not shown), indicating that other yet to be identified mechanisms may involve in this process. Based on the established work model of TRAF3 suppressing NF-κB activity, our findings suggest that SGK1 protects TRAF3 against ubiquitination-mediated degradation and in turn restrains NF-κB activity in *P. gingivalis*–stimulated innate immune cells.

### Silencing of TRAF3 or overexpression of c-Jun mimics the effect of SGK1 inhibition on the production of inflammatory cytokines

**Figure 5:**
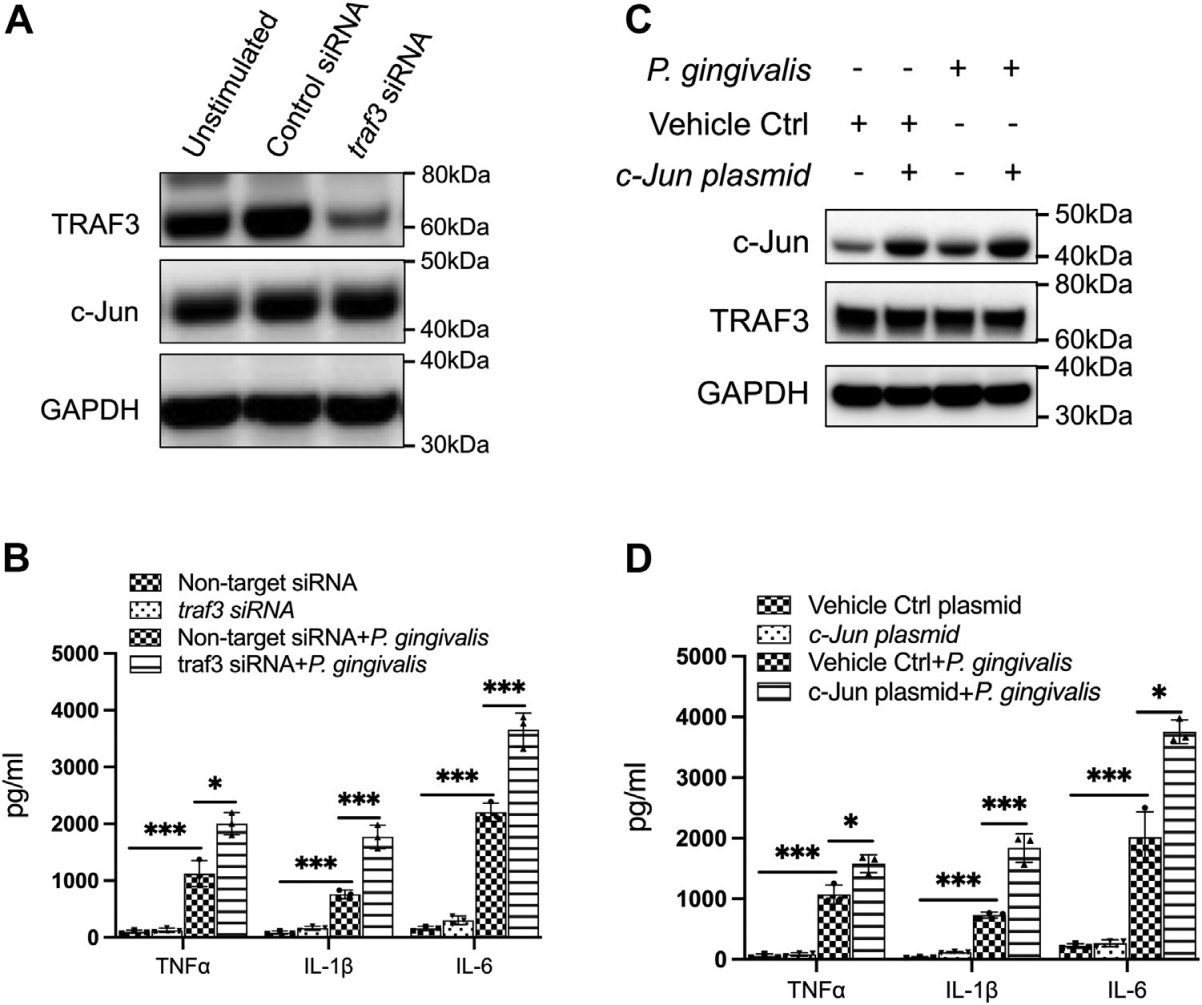
**siRNA-mediated silencing of *traf3* or overexpression of c-Jun mimics the effect of SGK1 deficiency on *Porphyromonas gingivalis*–induced inflammatory cytokines.** BMDMs were pretreated with prevalidated specific *traf3* siRNA or a plasmid encoding c-Jun for 48 h, then stimulated with *P. gingivalis* for 12 h followed by the inflammatory cytokine assay with ELISA. A scramble siRNA and a vehicle plasmid were used as controls. *A* and *C*, the whole cell lysates were harvested to test the transfection efficacy of *traf3* siRNA and expression of c-Jun with Western blots, showing that the alteration of c-Jun was irrelevant to the expression of TRAF3, and vice versa. GAPDH was used as a loading control in Western blotting. *B* and *D*, ELISA showing the production of proinflammatory cytokines, including TNFα, IL-1β, and IL-6. All results are the average of at least three independent experiments. Error bars represent SD. ∗ and ∗∗∗ represent *p* < 0.05 and *p* < 0.001, respectively. BMDM, bone marrow–derived macrophage; IL, interleukin; SGK1, serum- and glucocorticoid-regulated kinase 1; TNFα, tumor necrosis factor alpha.

TRAF3 has been demonstrated to negatively regulate the activity of NF-κB and thereby dictates secretion of TLR-mediated proinflammatory cytokines (36, 37, 52). Moreover, as a key component of AP-1 sites, c-Jun has also been demonstrated as a critical controller to the production of inflammatory cytokines (53, 54, 55). Since we have found that SGK1 inhibition or deficiency modulates expression of these two inflammatory regulators, we next examined whether SGK1 regulates *P. gingivalis*–mediated inflammatory cytokines through modulation of their expression. Using prevalidated specific siRNA, we found that silencing of TRAF3 significantly enhanced production of TNFα, IL-1β, and IL-6 in *P. gingivalis*–stimulated BMDMs, which exactly mimics the effect of SGK1 deficiency (Fig. 5, *A* and *B*). Moreover, overexpression of c-Jun resulted in a similar effect on the production of these proinflammatory cytokines (Fig. 5, *C* and *D*). Intriguingly, expression of c-Jun was not affected by siRNA-mediated gene silencing of TRAF3, and vice versa (Fig. 5, *A* and *C*), indicating SGK1 inhibition mediated reduction of TRAF3, and increase of c-Jun could be through distinct pathways in response to the challenge of *P. gingivalis*. These results suggest that SGK1 regulates *P. gingivalis*–induced inflammatory cytokine production possibly through a divergent modulation of both TRAF3 and c-Jun expression.

### SGK1 inhibition aggravates inflammatory immune responses and modulates expression of TRAF3 and c-Jun in a *P. gingivalis*–induced bone loss model

**Figure 6:**
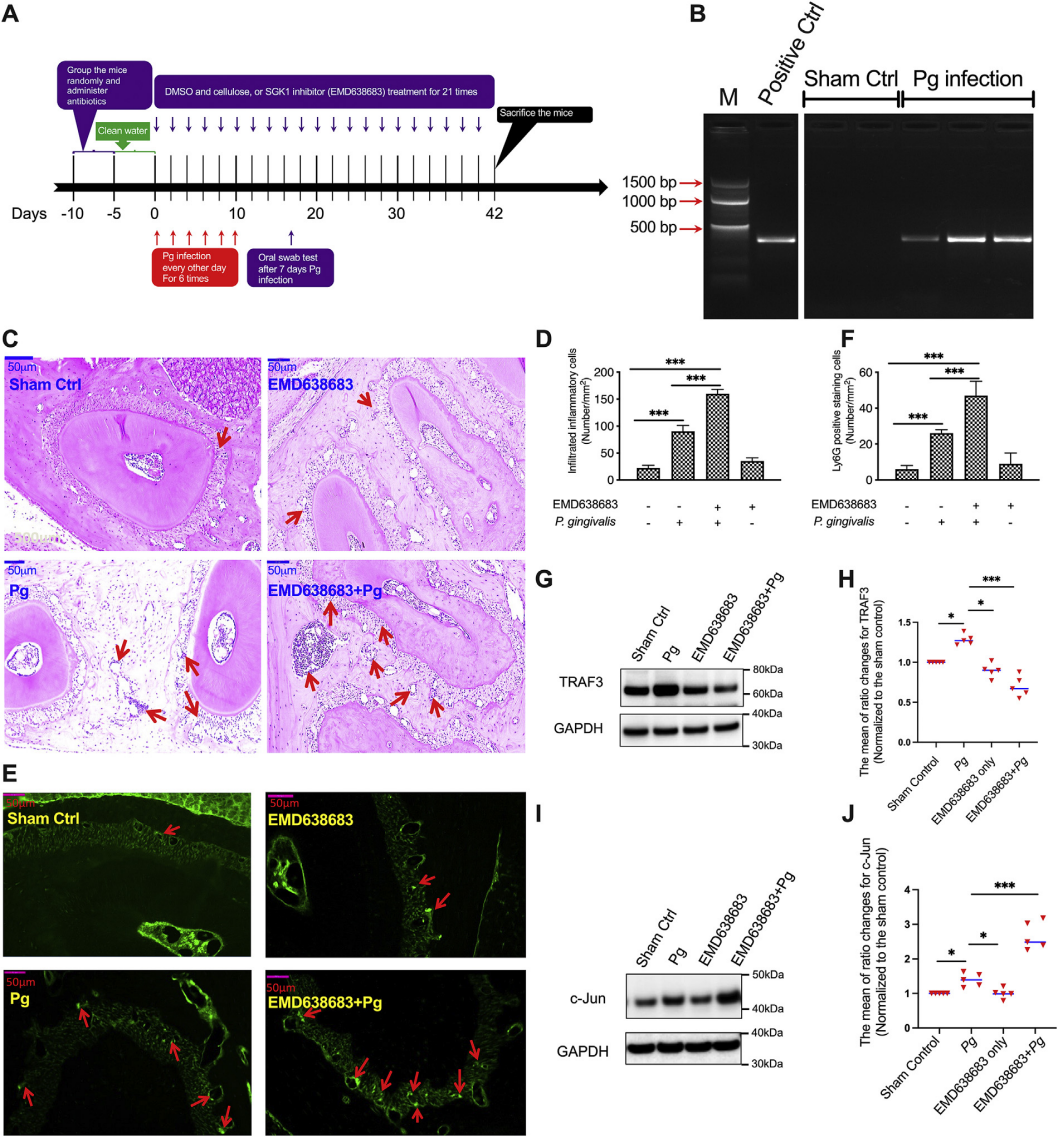
**SGK1 inhibition intensifies inflammatory cell infiltration and modulates expression of TRAF3 and c-Jun in *Porphyromonas gingivalis*–infected gingival tissues.** The 10- to 12-week-old C57/B6 mice were divided randomly into one sham control group and three experimental groups (n = 5 per group). The sham control group was treated with cellulose and 0.01% DMSO. The experimental groups were treated with *P. gingivalis* only, *P. gingivalis* with SGK1 inhibitor EMD638683 (15 mg/kg), or inhibitor only. Swabbing samples from the mouse oral cavity were examined by quantitative nested PCR to confirm *P. gingivalis* infection. *A*, the schematic flowchart showing the procedure of *P. gingivalis*–induced mice bone loss model. *B*, representative electrophoresis images showing *P. gingivalis* DNA obtained from oral samples. *C* and *D*, representative images of HE staining showing that *P. gingivalis* significantly increases inflammatory cell infiltration in gingival tissues, and SGK1 inhibition aggravates it. *E* and *F*, representative images of immunofluorescence staining showing that *P. gingivalis* increases infiltration of neutrophils, representing by the expression of Ly6G in gingival tissues (*E*), which is significantly increased with EMD638683 treatment (*F*). *G*–*J*, representative immunoblot images showing that EMD638683 significantly decreases expression of TRAF3 (*G* and *H*) but increases expression of c-Jun (*I* and *J*) in mice gingival tissues (n = 5 mice). *H* and *J*, the mean of ratio changes for TRAF3 and c-Jun, which were normalized to the sham control mice from each different group. The blots shown above are representative of five biological replicates. Error bars represent SD. ∗ and ∗∗∗ represent *p* < 0.05 and *p* < 0.001, respectively. DMSO, dimethyl sulfoxide; SGK1, serum- and glucocorticoid-regulated kinase 1; TRAF, tumor necrosis factor receptor–associated factor.

*P. gingivalis*–induced dysregulation of inflammatory responses has been demonstrated as a key factor in the initiation and progression of periodontitis (5, 41, 56). Since we found that SGK1 inhibition increases TNFα, IL-6, and IL-1β expression in *P. gingivalis*–stimulated immune cells, we next investigated whether SGK1 inhibition exacerbates the severity of periodontal bone loss using a mouse model of *P. gingivalis*–induced disease (3, 5) (Fig. 6*A*). We first utilized nested quantitative PCR (qPCR) to test the efficiency of *P. gingivalis* infection, represented by the amount of DNA recovered from the oral cavity. We found that *P. gingivalis* was successfully colonized in those infected mice (Fig. 6*B*). As overwhelming neutrophil and macrophage accumulation are critical to the development of periodontitis (57, 58) and our *in vitro* data showed SGK1 inhibition enhances production of MIP-2, we next examined whether SGK1 inhibition affects inflammatory cell infiltration by staining tissue sections with H&E (Fig. 6, *C* and *D*). We found that compared with no infection, *P. gingivalis* infection increased the infiltration of inflammatory cells in mice (Fig. 6*D*). Furthermore, the number of inflammatory cells in the gingival tissue of SGK1 inhibitor–treated mice was significantly increased compared with that in mice without SGK1 inhibitor treatment (Fig. 6*D*). This result was further confirmed by fluorescence staining using a neutrophil surface marker, Ly6G. The data also suggested that the majority of the infiltrated cells were neutrophils (Fig. 6, *E* and *F*). Since we found that SGK1 regulated the production of inflammatory cytokines by modulating expression of TRAF3 and c-Jun in cultured cells, we next examined their expression in mice gingival tissues. As expected, inhibition of SGK1 substantially reduced expression of TRAF3 (Fig. 6, *G* and *H*) but enhanced c-Jun (Fig. 6, *I* and *J*) in *P. gingivalis*–infected gingival tissues, suggesting SGK1 indeed regulates TRAF3 and c-Jun upon the challenge of *P. gingivalis*.

**Figure 7:**
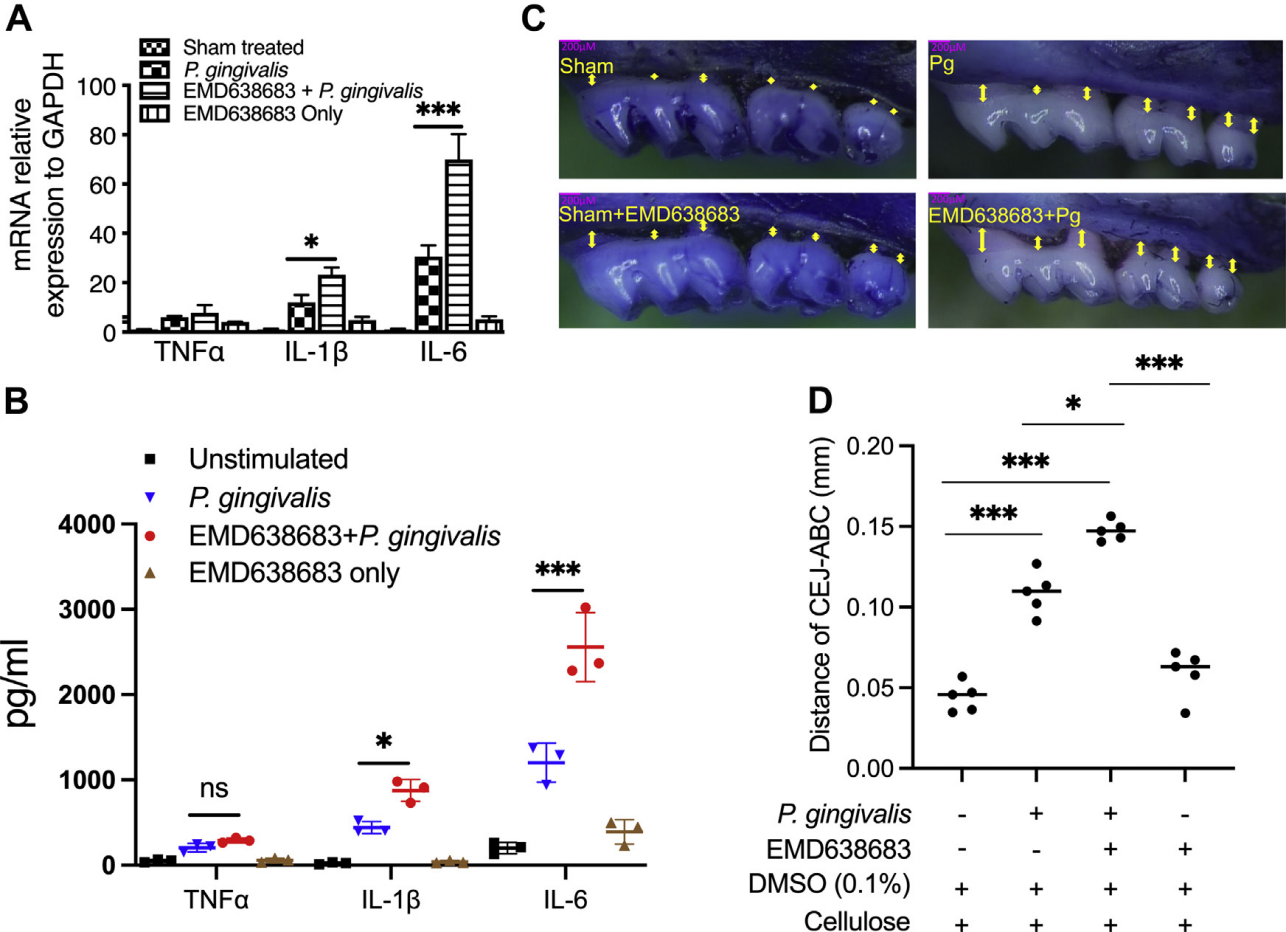
**SGK1 inhibition exacerbates *Porphyromonas gingivalis*–infection induced alveolar bone loss in a mouse model.***A*, quantitative RT–PCR showing that SGK1 inhibition significantly enhances secretion of inflammatory cytokines in mice gingival tissue (n = 5). *B*, ELISA showing the levels of inflammatory cytokines in mouse serum. *C*, representative images of bone loss using methylene blue/eosin staining and quantified under microscope. *D*, quantification of alveolar bone loss represented by the distance from the CEJ to the ABC. Data are presented as the mean CEJ–ABC distance in millimeters ± SD (n = 5 mice per group). Error bars represent the SD. ∗*p* < 0.05, ∗∗∗*p* < 0.001. Data represent the arithmetic mean ± SD of three independent experiments. ABC, alveolar bone crest; CEJ, cementoenamel junction; SGK1, serum- and glucocorticoid-regulated kinase 1.

Given that the production of proinflammatory cytokines lies at the heart of periodontal diseases, we next utilized qRT–PCR and ELISA to examine the mRNA levels of TNFα, IL-6, and IL-1β in gingival tissues and related protein levels in mouse serum. We found that IL-6 and IL-1β were significantly enhanced in SGK1 inhibitor–treated *P. gingivalis*–infected mice compared with mice infected with *P. gingivalis* only, but no substantial differences were observed in the amount of TNFα (Fig. 7, *A* and *B*). This discrepancy between *in vitro* and *in vivo* response to SGK1 inhibition may be due to multiple factors, such as the phase of inflammation, differential dynamics of cytokine synthesis, variant resistance to *P. gingivalis*–induced tolerance, mutual regulation of cytokines, and importantly, the different components of samples, indicating that complicated regulatory mechanisms are involved in the progression of chronic inflammation. In addition, *P. gingivalis* infection led to significant bone loss, and SGK1 inhibition aggravated the severity of the bone loss as determined through the measurement of the cementoenamel junction–alveolar bone crest distance (Fig. 7, *C* and *D*). These results demonstrate that inhibition of SGK1 promotes *P. gingivalis*–induced inflammatory responses and aggravates periodontal bone loss in a mouse model, suggesting SGK1 could be an interventional target for protecting against periodontal bone loss by restraining the oral bacteria–induced inflammatory response. Future studies examining the expression of SGK1, TRAF3, and c-Jun and their relevance to the predisposition to periodontitis will unravel the potential therapeutic significance of these protein kinases.

## Discussion

**Figure 8:**
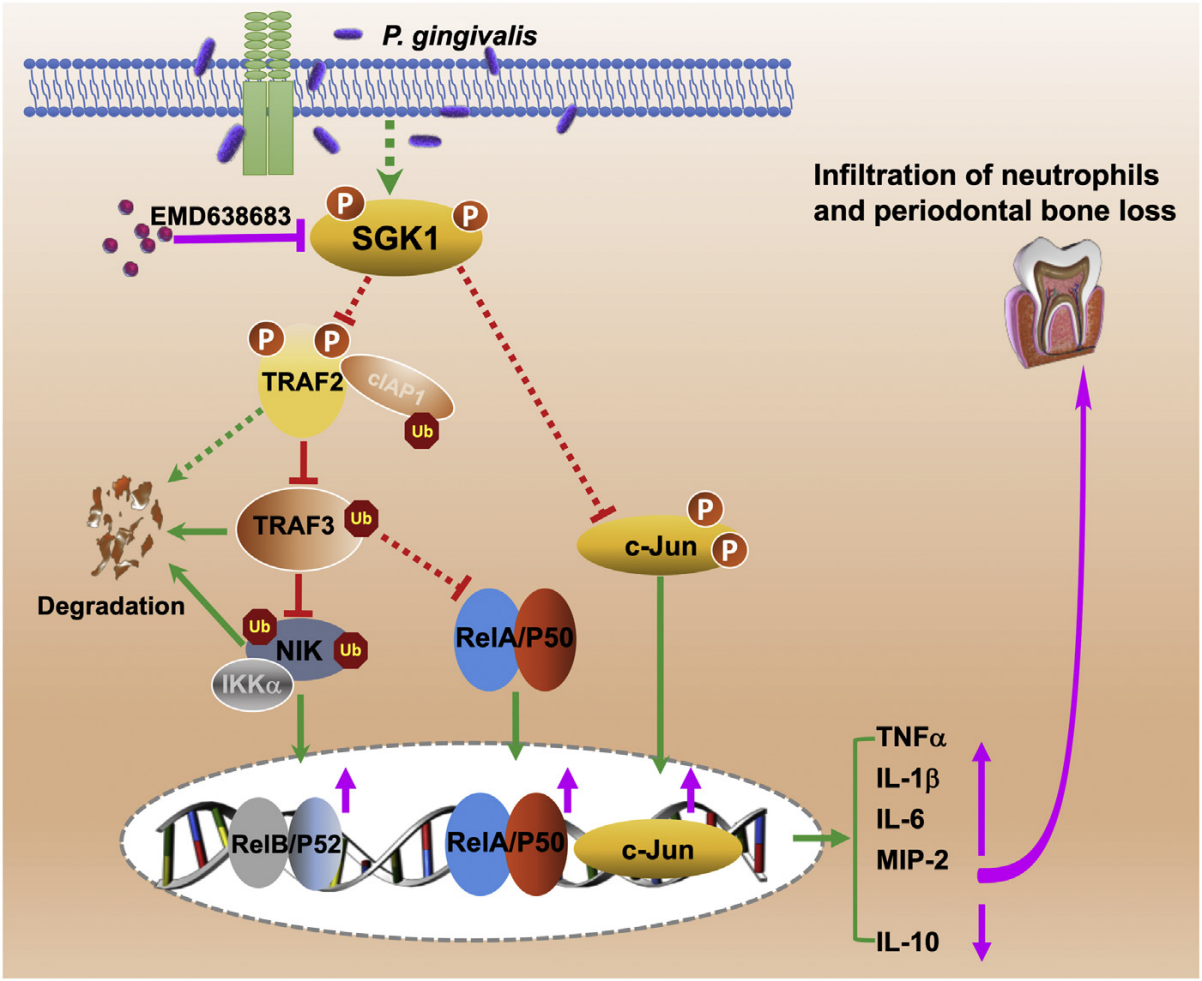
**Schematic model of SGK1 restrains *Porphyromonas gingivalis*–mediated inflammatory responses through modulation of TRAF2/3 and c-Jun.** While *P. gingivalis* infection activates proinflammatory signaling (not shown here) and thus induces the production of proinflammatory cytokines, SGK1 is concomitantly phosphorylated and in turn suppresses the expression of c-Jun, the phosphorylation of TRAF2, and thereby limits ubiquitination-mediated TRAF3 degradation, which promotes degradation of NIK and thus restrains NF-κB activity (for both the canonical and noncanonical pathways) and subsequent proinflammatory cytokines. In contrast, SGK1 inhibition or deficiency (*pink*) enhances the expression of c-Jun, the phosphorylation of TRAF2, and thereby promotes degradation of TRAF3, upregulates activity of NIK/IKK axis, and thus boosts activity of NF-κB (*pink*), which would increase the production of proinflammatory cytokines (*pink*), and ultimately leading to increase of inflammatory cell infiltration and the consequent exacerbation of inflammation-induced alveolar bone loss. IKK, inhibitor of upstream IκB kinase; NIK, NF-κB–inducing kinase; SGK1, serum- and glucocorticoid-regulated kinase 1; TRAF, tumor necrosis factor receptor–associated factor.

In this study, we found that SGK1 inhibition enhances secretion of proinflammatory cytokines in cultured cells and aggravates inflammatory immune responses in a murine periodontal bone loss model. Moreover, we identified a novel SGK1-mediated signaling module to restrain *P. gingivalis*–induced inflammatory immune responses. SGK1 deficiency promotes TRAF3 degradation through upregulating phosphorylation of a ubiquitin E3 ligase, TRAF2, in *P. gingivalis*–stimulated innate immune cells. A decrease of TRAF3, combined with an increase of c-Jun, could boost activation of NF-κB and thereby aggravate *P. gingivalis*–mediated inflammatory immune responses. Therefore, this study will be the first to define the functional role of the SGK1–TRAF2/3–c-Jun/NF-κB signaling axis in regulation of *P. gingivalis*–mediated host inflammatory immune responses (Fig. 8). Given that TRAF member–mediated ubiquitinations are extensively involved in the modulation of innate immune responses, our findings will provide more insights on understanding the interaction between serine/threonine kinase and ubiquitin ligases, exemplified by SGK1 and TRAF2/3, as well as their function in oral bacteria–mediated inflammation. In addition, these findings will provide an alternative strategy to resolve the overwhelming inflammation through augmenting the activity of anti-inflammatory pathways and aid in development of novel interventional targets with relevance for chronic and debilitating inflammatory diseases.

Previous studies demonstrated that SGK1 phosphorylates and inactivates Nedd4-2, a HECT (homologous to E6AP C terminus) ubiquitin E3 ligase (39, 59, 60). Apart from HECT E3s, there are two other main types of E3s including RING and RBR E3s with vastly different mechanisms of ubiquitin transfer, that is, HECT E3s directly transfer the ubiquitin to substrates, whereas RING ligases function as scaffolds and coordinate transfer together with the E2 enzyme (61). While our and other previous studies showed that Nedd4-2 is necessary for inflammatory responses through controlling the activity of P38 and NF-κB signaling pathways (5, 22, 38), it remains unknown whether SGK1 can modulate the activity of other ubiquitin ligases, apart from HECT E3s, and function in the regulation of inflammation. In this study, our results show that SGK1 deficiency promotes phosphorylation of a prototypical RING E3 ligase, TRAF2, and consequently controls degradation of TRAF3 in response to *P. gingivalis* challenge, suggesting SGK1 may act as a hub in regulation of different types of ubiquitin ligases. Further studies focusing on the interactions between SGK1 activity and other ubiquitin ligases will provide more insights on this point and define the specific regulatory function of TRAF2 and TRAF3 in the inflammatory milieu of periodontal tissues.

TRAF2 and TRAF3 were reported as multiple-faced regulators in NF-κB–mediated inflammatory responses (62). Through binding to cIAPs, they can form a complex and restrain the activation of noncanonical NF-κB signaling in a ubiquitination-dependent manner (35). Moreover, TRAF2 was also found to collaborate with TRAF3 and suppress TLR-mediated proinflammatory cytokine production. Deficiency of either TRAF2 or TRAF3 leads to an increase of TLR-mediated proinflammatory cytokines *in vitro* and *in vivo* (36, 37), which was consolidated by a recent study showing that TRAF2 knockout promotes activation of NF-κB and elevates inflammation responses in skin tissue (33). Intriguingly, we found that SGK1 deficiency–mediated decrease of TRAF3 promotes the activity of canonical as well as noncanonical NF-κB activity in response to *P. gingivalis* challenge. Regarding this point, previous studies (36, 37, 63) have reported that TRAF3 deletion could also promote canonical NF-κB activity through modulation of different NF-κB members in different cells. Thus, our results suggest that TRAF3 may function as a suppressor for both canonical and noncanonical NF-κB activity, at least in response to *P. gingivalis* challenge. Notably, recent studies reported the opposite roles of TRAF3 in regulating RNA and DNA virus–induced type I interferon production (64). Given type I interferon and IL-10 is pivotal for TRAF3 to suppress inflammatory responses (65, 66), the anti-inflammatory effect of TRAF3 is likely dependent on the specific contexts, and therefore, more future investigations will warrant the regulatory effect of TRAF3 on oral bacteria–mediated inflammatory immune responses.

Apart from regulation on *P. gingivalis*–induced inflammatory cytokines (67), activation of c-Jun is key for *P. gingivalis*–induced alveolar bone loss through controlling expression of receptor activator of NF-κB ligand (68). In this study, our results show that SGK1 inhibition significantly increases expression of c-Jun, yet we did not elucidate how SGK1 regulates the expression of c-Jun. SGK1 has been reported to phosphorylate GSK3β under a variety of contexts (69). Our previous study has demonstrated that GSK3β phosphorylation leads to phosphorylation of c-Jun at Thr239 in lipopolysaccharide-stimulated BMDMs (70). Given phosphorylation of c-Jun at Thr239 leads to its degradation, it is reasonable to speculate that SGK1 may enhance phosphorylation of GSK3β and subsequent c-Jun phosphorylation at Thr239 and thereby decreases expression of c-Jun in *P. gingivalis*–stimulated BMDMs. Thus, it would be very interesting to identify the downstream substrates of SGK1 under different contexts, which could provide more insights on the regulatory mechanisms of SGK1. On the other hand, we found that *P. gingivalis* infection leads to a rapid decrease of c-Jun (data not shown), indicating the gingipains secreted by *P. gingivalis* may also be involved in the regulation of c-Jun regulation. Further investigation on the mechanism of *P. gingivalis* induced decrease of c-Jun, and the role of gingipain in this process will reveal how this oral bacterium modulates expression of c-Jun kinetically and thereby exploits host inflammatory immune responses.

Although the detailed mechanism of how SGK1 differentially impacts phosphor-TRAF2 and total TRAF3 is yet to be known, based on the model of TRAF2-mediated TRAF3 degradation in previous studies (46, 71), it is possible that inhibition of SGK1 promotes TRAF2 phosphorylation, which catalyzes K63 polyubiquitination of cIAPs, and in turn targets TRAF3 for degradation by promoting its K48 polyubiquitination. In addition, *P. gingivalis* has been shown to impact the activity of a ubiquitin-editing enzyme A20 and other E3 ligases such as Smurf1 (5, 40). Infection with *P. gingivalis* dramatically increases A20 expression in innate immune cells, which subsequently interacts with TRAF1 and TRAF2 to form a ubiquitin-editing complex to prevent K63 polyubiquitin chain synthesis by TRAF6. This leads to inhibitor of upstream IκB kinase inhibition and NF-κB suppression (71, 72). Combined with our current findings about SGK1 protects against TRAF3 degradation, it is reasonable to speculate that *P. gingivalis* may employ a two-pronged strategy to induce anti-inflammatory responses: one is through activating A20 that will decrease K63 polyubiquitination and reduce TRAF6 migration, whereas the other is to activate SGK1, diminishes TRAF2 phosphorylation, and thus limit TRAF3 degradation, both eventually restraining the activity of NF-κB and consequent inflammatory immune responses. In addition, we cannot exclude the possibility that SGK1 is interconnected with the signaling of A20, either by overlapping or integrating with signaling to modify downstream TRAFs, which subsequently suppresses inflammatory responses. Therefore, further investigation on *P. gingivalis* infection–mediated global alteration of E3s will warrant the critical regulatory function of SGK1 in periodontal inflammation and open a new avenue to elucidate the pathogenesis of periodontitis.

In this study, our results have shown a discrepancy in TNFα production between *in vitro* and *in vivo* assays. SGK1 inhibition significantly enhanced the production of TNFα, IL-1β, and IL-6 in *P. gingivalis*–stimulated cultured cells, yet only IL-6 and IL-1β were significantly increased in the gingival tissue from *P. gingivalis*–infected mice. This discrepancy might be due to the phase of inflammation, differential dynamics of cytokines synthesis, variant resistance to *P. gingivalis*–induced tolerance, and most importantly, the different components of samples. In our animal model, the gingival tissues for cytokine analysis were collected from mice postinfection with *P. gingivalis* for 42 days, which is approximately equal to 6 years of infection in humans. Compared with the cultured cells challenged with *P. gingivalis* for 12 h, it is not surprising to see the different phase of inflammation leads to the different levels of TNFα. Moreover, TNFα is produced more quickly and lasts a shorter time as compared with IL-6 in many inflammation models (73), which could be another reason for being unable to observe SGK1 inhibition of TNFα from gingival tissue. In addition, the difference of the samples we used *in vitro* and *in vivo* could also be a major reason for this discrepancy. Unlike an *in vitro* monoculture, gingival tissue includes not only monocytes and macrophages but also neutrophils, adaptive immune cells, epithelial cells, fibroblasts, and other cells. Although we did not examine the function of SGK1 in adaptive immune cells from gingival tissue, the opposite regulatory function of SGK1 in innate and adaptive immune cells has been reported (22, 23, 27) and thus possibly affects the final readout of the net effect of SGK1 on the levels of inflammatory cytokines. Because of the limited size of murine gingival tissue, it is difficult for us to isolate monocytes or macrophages to test the possible distinct function of SGK1 in single types of immune cells. Future research focusing on the production of inflammatory cytokines at different phases of *P. gingivalis* infection, and using single-cell sequencing analysis from pooled tissues or cervical lymph nodes, will likely provide more interesting information.

In summary, we have demonstrated for the first time that SGK1 restrains *P. gingivalis*–mediated inflammatory immune responses through modulation of TRAF2/3 and c-Jun. Moreover, we have mapped out details of this novel signaling axis. SGK1 activation dampens phosphorylation of TRAF2 and reduces degradation of TRAF3 in a ubiquitination-dependent manner. With the addition of the suppressed c-Jun, activation of SGK1 suppresses the activity of NF-κB and subsequent inflammatory responses. Our *in vivo* data using a *P. gingivalis*–induced alveolar bone loss model also confirmed the anti-inflammatory role of SGK1-TRAF2/3–c-Jun/NF-κB signaling axis, suggesting that targeting SGK1-mediated TRAF signaling, or the expression of c-Jun, could be a novel strategy to manipulate the intensity and direction of inflammation and thus benefit patients with inflammatory diseases relevant beyond periodontitis.

## Experimental procedures

### Mice, bacteria, and reagents

*LysM*-Cre^+^*sgk1*^*fl/fl*^ mice were generated by crossing *LysM*-driven expression of Cre C57BL/6 mice (from The Jackson Laboratory) to floxed (loxP flanked) *sgk1* mice (provided by Dr Alexander; Dartmouth Medical School). Control mice were negative littermates from this breeding. All the mice were housed in a specific pathogen-free facility at Virginia Commonwealth University (VCU), and the VCU Institutional Animal Care and Use Committee approved all animal protocols. All efforts were made to minimize the number of mice used and to prevent animal distress, pain, and injury. Carbon dioxide (CO_2_) was used for euthanasia of mice. *P. gingivalis* 33277 was from American Type Culture Collection and cultured anaerobically in trypticase soy broth supplemented with yeast extract (1 mg/ml), hemin (5 μg/ml), and menadione (1 μg/ml). *S. gordonii* DL1 (from American Type Culture Collection) and *S. sanguinis* SK36 (from Dr Kitten’s laboratory) were grown at 37 °C in Anoxomat jars (Spiral Biotech) under microaerobic conditions (7% H_2_, 7% CO_2_, 80% N_2_, and 6% O_2_) in brain–heart infusion (Bacto) broth. Ultrapure lipopolysaccharide (1 μg/ml) from *Escherichia coli 0111:B4* was from Invivogen. Phospho-SGK1 antibodies were from Santa Cruz Biotechnology. Total SGK1 antibodies were from Proteintech. Anti-NF-κB P52 and anti-NIK antibodies were from Abcam. All other antibodies were from Cell Signaling Technology. The SGK1 inhibitor, EMD638683, was from MedChem Express and has been shown to be specific for SGK1 without discernible effects on a panel of 68 other kinases (74) and the survival of innate immune cells with the concentration of 15 μM that we used in this study (22). The proteasome inhibitor MG-132 (also known as carbobenzoxyl-l-leucyl-l-leucyl-l-leucinal; ZLLL-CHO) was from SelleckChem. Nontargeting pools of siRNA and a mixture of four prevalidated siRNA specific for *sgk1* and *traf3* were from GE Healthcare Dharmacon. The plasmid encoding c-Jun and the vehicle plasmid pCMV-Tag2A, both were from Addgene. Mouse TNFα, IL-6, and IL-1β cytokine ELISA kits were purchased from BioLegend. MIP2/CXCL2 kit was from R&D Systems. RNeasy Mini and RNase-Free DNase Set were from QIAGEN. High-Capacity complementary DNA reverse transcription kit and TaqPath qPCR master mix were from Applied Biosystems.

### Preparation of human monocytes, BMDMs, and oral epithelial cells

Peripheral blood mononuclear cells were obtained from the venous blood of healthy donors as per protocols approved by the University of Louisville, Institutional Review Board. Monocytes were isolated by negative selection using the human monocyte isolation kit II from Miltenyi Biotec, Inc. The purity of monocytes was routinely >90%, as determined by flow cytometry using an FITC-labeled anti-CD14 antibody. BMDMs were generated from femoral and tibial bone marrow cells as previously described (75). Briefly, bone marrow was flushed from the femur and tibiae of 8-week-old *LysM*-Cre^+^ *sgk*^*fl/fl*^ or the littermate control mice using sterile Hanks’ balanced salt solution and homogenized by passage through an 18.5-gauge syringe repeatedly. The cells were washed in PBS, centrifuged at 1500 rpm for 5 min, and resuspended in RPMI1640 medium supplemented with 10% fetal bovine serum, 50 μM 2-mercaptoethanol, 1 mM sodium pyruvate, 2 mM l-glutamine, 20 mM Hepes, 50 units/ml penicillin, 50 μg/ml streptomycin, and 30% L929 culture supernatant. Nonadherent cells were collected after 24 h and cultured for 7 days in Costar ultra-low attachment polystyrene culture dishes with a medium change on day 4. Macrophages were about 95% F4/80^+^/CD11b^+^ as determined by flow cytometry and ready for further experiments. Mice primary oral epithelial cells were purchased from Lifeline Cell Technology and maintained in our laboratory according to the manufacturer’s procedure.

### siRNA and plasmid transfection, cytokine assay, and Western blots

siRNA and plasmid transfections in human monocytes were carried out by electroporation using a Nucleofector device according to the proposed protocol. Briefly, after isolation of monocytes, 4 × 10^6^ monocytes were resuspended in 100 μl of Nucleofector solution (Human Monocytes Nucleofector kit; Amaxa) together with 2 μg of a GFP-coding plasmid (pCMV-GFP) and 2 μg of siRNA duplexes for each target. After electroporation, 400 μl of prewarmed M-199 containing 10% fetal calf serum was added to the cuvette, and the cells were transferred into culture plates containing prewarmed M-199 with 10% fetal calf serum. Cells were challenged at the optimal time of gene silencing (48 h post-transfection). BMDMs were transfected with nontargeting control siRNA, siRNA-*traf3*, pCMV-Tag2-c-Jun, or pCMV-Tag2 (empty vector control) using Lipofectamine RNAiMAX or Lipofectamine LTX (Invitrogen) following the manufacturer’s protocol. After transfection, the cells were directly seeded in either 96-well or 6-well plates. The levels of TRAF3 and c-Jun were assessed by Western blots. Cells were lysed in radioimmunoprecipitation assay buffer containing phosphatase inhibitors for Western blot assays. Images were acquired using the G:Box Chemi XXI (Syngene). For cytokine assays, cell-free supernatants were collected after stimulation with *P. gingivalis* 12 h instead of 24 h to minimize gingipain-induced proteolytic cleavage of cytokines. For the systemic cytokine concentration assays, mouse whole blood was collected by cardiac puncture after their euthanization, 42 days from the initial infection, and cell-free serum was isolated according to the protocol described previously (3). Cytokine concentration was determined by ELISA following the manufacturer’s instructions (eBioscience).

### RNA isolation, real-time qRT–PCR, and flow cytometry

Total RNA was isolated using RNeasy Mini kit (QIAGEN) with RNase-Free DNase Set according to the manufacturer’s instructions. RNA samples were reverse transcribed with the High-Capacity complementary DNA reverse transcription kit. Real-time qPCR analysis was performed using specific primers for mouse *sgk1* (Mm00441380), *tnfα* (Mm00443258_m1), *il-1β* (Mm00434228_m1), and *il-6* (Mm00446190_m1) in an Applied Biosystems 7500 system using TaqPath qPCR master mix. Relative levels of gene expression were determined using *gapdh* (Mm99999915_g1) as the control (76). For IC staining IL-10, human monocytes were either untreated or treated with *P. gingivalis* for 8 h followed by the treatment with brefeldin A from eBioscience for 6 h. Then cells were fixed with IC fixation buffer for 30 min, followed by washing twice with permeabilization buffer and incubation with phycoerythrin-conjugated IL-10 for 30 min. Stained cells were then washed and analyzed on an LSR Fortessa flow cytometer (BD Biosciences). All the reagents for flow cytometry assays are from eBioscience, and all the data were analyzed with the FlowJo software (BD Biosciences).

### *P. gingivalis*–induced periodontal inflammation model and immunohistochemistry

The endogenous oral microbiota were suppressed in 10- to 12-week-old C57/B6/J mice by sulfamethoxazole (800 μg/ml) and trimethoprim (400 μg/ml) provided *ad libitum* in water for 5 days. The mice then received pure drinking water for 5 days. Alveolar bone loss was induced by oral infection with 1 × 10^9^ colony-forming unit of *P. gingivalis* suspended in 100 μl of PBS with 2% carboxymethylcellulose. Infections were performed six times at every other day. An experimental group was also intraperitoneally administered EMD638683 (15 mg/kg) with the infection every other day until euthanization. Sham-infected and vehicle control mice were also established. The mice were euthanized with CO_2_ and cervical dislocation 42 days after the final infection. Maxillary gingiva from mice upper jaws were harvested with a half used for RT–PCR assay and the other half for Western blot assay. The fresh gingival tissues were immersed in RNAlater (Ambion; catalog no.: AM7020) or radioimmunoprecipitation assay buffer with protease and phosphatase inhibitors (MilliporeSigma; catalog nos.: P8340 and P0044) (1:100 dilution) and then stored at −20 °C for further RT–PCR or Western blot assay, respectively. For Western blot assay, the gingival tissue samples were run on five separate gels, each included four individual samples, and every sample was from each different experimental group, respectively. After Western blotting assay, we calculated the mean of ratio changes for TRAF3 and c-Jun, which were normalized to the sham control mice from each different group. The lower jaws of the mice were fixed in 4% paraformaldehyde, decalcified in Immunocal solution for 15 days, and embedded in paraffin wax for immunofluorescence assays. Alveolar bone loss was measured in millimeters at 14 predetermined points per mouse on the maxillary molars of defleshed maxillae as the distance from the cementoenamel junction to the alveolar bone crest. Bone loss was visualized by methylene blue/eosin staining and quantified using a Nikon SMX 800 dissecting microscope (40×) fitted with a Boeckeler VIA-170K video image marker measurement system. The results were expressed as the mean with SD. The paraffin-embedded tissue blocks were freshly cut into 4 μm mesiodistal sections for subsequent immunostaining with H&E to evaluate inflammatory cell infiltration, and Alexa 488-conjugated antimouse Ly6G was used to measure the infiltration of neutrophils. At least five serial tissue sections from the same block were used to measure the positive area, and 20 adjacent fields of view from the same anatomic area of each tissue section were selected to count the total number of inflammatory cells.

### Statistical analyses

The statistical significance of differences between groups was evaluated by the ANOVA and the Tukey multiple comparison test using the InStat program (GraphPad Software, Inc). Differences between groups were considered significant at the level of *p* ≤ 0.05.

## Data availability

All the raw data used for figure generation and any additional information are available upon request from Huizhi Wang (wangh3@vcu.edu).

## Supporting information

This article contains supporting information.

## Conflict of interest

The authors declare that they have no conflicts of interest with the contents of this article.

## References

[1] Murray P.J., Smale S.T. (2012). Restraint of inflammatory signaling by interdependent strata of negative regulatory pathways. Nat. Immunol..

[2] Fullerton J.N., Gilroy D.W. (2016). Resolution of inflammation: a new therapeutic frontier. Nat. Rev. Drug Discov..

[3] Adamowicz K., Wang H., Jotwani R., Zeller I., Potempa J., Scott D.A. (2012). Inhibition of GSK3 abolishes bacterial-induced periodontal bone loss in mice. Mol. Med..

[4] Li Y., Mooney E.C., Holden S.E., Xia X.J., Cohen D.J., Walsh S.W. (2019). A20 orchestrates inflammatory response in the oral mucosa through restraining NF-kappaB activity. J. Immunol..

[5] Lu L., Yakoumatos L., Ren J., Duan X., Zhou H., Gu Z. (2020). JAK3 restrains inflammatory responses and protects against periodontal disease through Wnt3a signaling. FASEB J..

[6] Yang D., Li S., Duan X., Ren J., Liang S., Yakoumatos L. (2020). TLR4 induced Wnt3a-Dvl3 restrains the intensity of inflammation and protects against endotoxin-driven organ failure through GSK3beta/beta-catenin signaling. Mol. Immunol..

[7] Nakayama M., Inoue T., Naito M., Nakayama K., Ohara N. (2015). Attenuation of the phosphatidylinositol 3-kinase/Akt signaling pathway by Porphyromonas gingivalis gingipains RgpA, RgpB, and Kgp. J. Biol. Chem..

[8] Eke P.I., Dye B.A., Wei L., Thornton-Evans G.O., Genco R.J., CDC Periodontal Disease Surveillance Workgroup: James Beck, G.D.R.P. (2012). Prevalence of periodontitis in adults in the United States: 2009 and 2010. J. Dent Res..

[9] Bostanci N., Belibasakis G.N. (2012). Porphyromonas gingivalis: an invasive and evasive opportunistic oral pathogen. FEMS Microbiol. Lett..

[10] Gibson F.C., Ukai T., Genco C.A. (2008). Engagement of specific innate immune signaling pathways during Porphyromonas gingivalis induced chronic inflammation and atherosclerosis. Front. Biosci..

[11] Hajishengallis G., Krauss J.L., Liang S., McIntosh M.L., Lambris J.D. (2012). Pathogenic microbes and community service through manipulation of innate immunity. Adv. Exp. Med. Biol..

[12] Berker E., Kantarci A., Hasturk H., Van Dyke T.E. (2013). Blocking proinflammatory cytokine release modulates peripheral blood mononuclear cell response to Porphyromonas gingivalis. J. Periodontol..

[13] Hajishengallis G. (2014). Immunomicrobial pathogenesis of periodontitis: keystones, pathobionts, and host response. Trends Immunol..

[14] Hajishengallis G. (2015). Periodontitis: from microbial immune subversion to systemic inflammation. Nat. Rev. Immunol..

[15] Hajishengallis G., Lamont R.J. (2014). Breaking bad: manipulation of the host response by Porphyromonas gingivalis. Eur. J. Immunol..

[16] Cullinan M.P., Ford P.J., Seymour G.J. (2009). Periodontal disease and systemic health: current status. Aust. Dent. J..

[17] Meyer M.S., Joshipura K., Giovannucci E., Michaud D.S. (2008). A review of the relationship between tooth loss, periodontal disease, and cancer. Cancer Causes Control.

[18] Stafford P., Higham J., Pinnock A., Murdoch C., Douglas C.W., Stafford G.P. (2013). Gingipain-dependent degradation of mammalian target of rapamycin pathway proteins by the periodontal pathogen Porphyromonas gingivalis during invasion. Mol. Oral Microbiol..

[19] Zhou Y., Sztukowska M., Wang Q., Inaba H., Potempa J., Scott D.A. (2015). Noncanonical activation of beta-catenin by Porphyromonas gingivalis. Infect. Immun..

[20] Lu X., Crowley S.D. (2018). Inflammation in salt-sensitive hypertension and renal damage. Curr. Hypertens. Rep..

[21] Lang F., Gorlach A., Vallon V. (2009). Targeting SGK1 in diabetes. Expert Opin. Ther. Targets.

[22] Ren J., Han X., Lohner H., Liang R., Liang S., Wang H. (2021). Serum- and glucocorticoid-inducible kinase 1 promotes alternative macrophage polarization and restrains inflammation through FoxO1 and STAT3 signaling. J. Immunol..

[23] Wu C., Yosef N., Thalhamer T., Zhu C., Xiao S., Kishi Y. (2013). Induction of pathogenic TH17 cells by inducible salt-sensing kinase SGK1. Nature.

[24] Matthias J., Maul J., Noster R., Meinl H., Chao Y.Y., Gerstenberg H. (2019). Sodium chloride is an ionic checkpoint for human TH2 cells and shapes the atopic skin microenvironment. Sci. Transl. Med..

[25] Garcia-Martinez J.M., Alessi D.R. (2008). mTOR complex 2 (mTORC2) controls hydrophobic motif phosphorylation and activation of serum- and glucocorticoid-induced protein kinase 1 (SGK1). Biochem. J..

[26] Di Cristofano A. (2017). SGK1: the dark side of PI3K signaling. Curr. Top. Dev. Biol..

[27] Zhou H., Gao S., Duan X., Liang S., Scott D.A., Lamont R.J. (2015). Inhibition of serum- and glucocorticoid-inducible kinase 1 enhances TLR-mediated inflammation and promotes endotoxin-driven organ failure. FASEB J..

[28] Heikamp E.B., Patel C.H., Collins S., Waickman A., Oh M.H., Sun I.H. (2014). The AGC kinase SGK1 regulates TH1 and TH2 differentiation downstream of the mTORC2 complex. Nat. Immunol..

[29] Yang M., Zheng J., Miao Y., Wang Y., Cui W., Guo J. (2012). Serum-glucocorticoid regulated kinase 1 regulates alternatively activated macrophage polarization contributing to angiotensin II-induced inflammation and cardiac fibrosis. Arterioscler. Thromb. Vasc. Biol..

[30] Dhillon B., Aleithan F., Abdul-Sater Z., Abdul-Sater A.A. (2019). The evolving role of TRAFs in mediating inflammatory responses. Front. Immunol..

[31] Xie P. (2013). TRAF molecules in cell signaling and in human diseases. J. Mol. Signal..

[32] Deshaies R.J., Joazeiro C.A. (2009). RING domain E3 ubiquitin ligases. Annu. Rev. Biochem..

[33] Etemadi N., Chopin M., Anderton H., Tanzer M.C., Rickard J.A., Abeysekera W. (2015). TRAF2 regulates TNF and NF-kappaB signalling to suppress apoptosis and skin inflammation independently of Sphingosine kinase 1. Elife.

[34] Skaug B., Jiang X., Chen Z.J. (2009). The role of ubiquitin in NF-kappaB regulatory pathways. Annu. Rev. Biochem..

[35] Yang X.D., Sun S.C. (2015). Targeting signaling factors for degradation, an emerging mechanism for TRAF functions. Immunol. Rev..

[36] Jin J., Xiao Y., Hu H., Zou Q., Li Y., Gao Y. (2015). Proinflammatory TLR signalling is regulated by a TRAF2-dependent proteolysis mechanism in macrophages. Nat. Commun..

[37] Zarnegar B., Yamazaki S., He J.Q., Cheng G. (2008). Control of canonical NF-kappaB activation through the NIK-IKK complex pathway. Proc. Natl. Acad. Sci. U. S. A..

[38] Grimsey N.J., Narala R., Rada C.C., Mehta S., Stephens B.S., Kufareva I. (2018). A tyrosine switch on NEDD4-2 E3 ligase transmits GPCR inflammatory signaling. Cell Rep..

[39] Debonneville C., Flores S.Y., Kamynina E., Plant P.J., Tauxe C., Thomas M.A. (2001). Phosphorylation of Nedd4-2 by Sgk1 regulates epithelial Na(+) channel cell surface expression. EMBO J..

[40] Maekawa T., Krauss J.L., Abe T., Jotwani R., Triantafilou M., Triantafilou K. (2014). Porphyromonas gingivalis manipulates complement and TLR signaling to uncouple bacterial clearance from inflammation and promote dysbiosis. Cell Host Microbe.

[41] Hajishengallis G., Chavakis T. (2021). Local and systemic mechanisms linking periodontal disease and inflammatory comorbidities. Nat. Rev. Immunol..

[42] Olsen I., Taubman M.A., Singhrao S.K. (2016). Porphyromonas gingivalis suppresses adaptive immunity in periodontitis, atherosclerosis, and Alzheimer's disease. J. Oral Microbiol..

[43] Olsen I., Yilmaz O. (2016). Modulation of inflammasome activity by Porphyromonas gingivalis in periodontitis and associated systemic diseases. J. Oral Microbiol..

[44] Murray J.T., Campbell D.G., Morrice N., Auld G.C., Shpiro N., Marquez R. (2004). Exploitation of KESTREL to identify NDRG family members as physiological substrates for SGK1 and GSK3. Biochem. J..

[45] Schmid E., Xuan N.T., Zahir N., Russo A., Yang W., Kuhl D. (2014). Serum- and glucocorticoid-inducible kinase 1 sensitive NF-kappaB signaling in dendritic cells. Cell. Physiol. Biochem..

[46] Workman L.M., Zhang L., Fan Y., Zhang W., Habelhah H. (2020). TRAF2 ser-11 phosphorylation promotes cytosolic translocation of the CD40 complex to regulate downstream signaling pathways. Mol. Cell. Biol..

[47] Lin W.J., Su Y.W., Lu Y.C., Hao Z., Chio I.I., Chen N.J. (2011). Crucial role for TNF receptor-associated factor 2 (TRAF2) in regulating NFkappaB2 signaling that contributes to autoimmunity. Proc. Natl. Acad. Sci. U. S. A..

[48] Kamynina E., Staub O. (2002). Concerted action of ENaC, Nedd4-2, and Sgk1 in transepithelial Na(+) transport. Am. J. Physiol. Renal Physiol..

[49] Pearce D. (2003). SGK1 regulation of epithelial sodium transport. Cell. Physiol. Biochem..

[50] Fotin-Mleczek M., Henkler F., Hausser A., Glauner H., Samel D., Graness A. (2004). Tumor necrosis factor receptor-associated factor (TRAF) 1 regulates CD40-induced TRAF2-mediated NF-kappaB activation. J. Biol. Chem..

[51] Zarnegar B.J., Wang Y., Mahoney D.J., Dempsey P.W., Cheung H.H., He J. (2008). Noncanonical NF-kappaB activation requires coordinated assembly of a regulatory complex of the adaptors cIAP1, cIAP2, TRAF2 and TRAF3 and the kinase NIK. Nat. Immunol..

[52] Lalani A.I., Luo C., Han Y., Xie P. (2015). TRAF3: a novel tumor suppressor gene in macrophages. Macrophage (Houst).

[53] de Haij S., Bakker A.C., van der Geest R.N., Haegeman G., Vanden Berghe W., Aarbiou J. (2005). NF-kappaB mediated IL-6 production by renal epithelial cells is regulated by c-Jun NH2-terminal kinase. J. Am. Soc. Nephrol..

[54] Schonthaler H.B., Guinea-Viniegra J., Wagner E.F. (2011). Targeting inflammation by modulating the Jun/AP-1 pathway. Ann. Rheum. Dis..

[55] Zhu C., Gagnidze K., Gemberling J.H., Plevy S.E. (2001). Characterization of an activation protein-1-binding site in the murine interleukin-12 p40 promoter. Demonstration of novel functional elements by a reductionist approach. J. Biol. Chem..

[56] Xu W., Zhou W., Wang H., Liang S. (2020). Roles of Porphyromonas gingivalis and its virulence factors in periodontitis. Adv. Protein Chem. Struct. Biol..

[57] Hroch M., Havlinova Z., Nobilis M., Chladek J. (2012). HPLC determination of arginases inhibitor N-(omega)-hydroxy-nor-L-arginine using core-shell particle column and LC-MS/MS identification of principal metabolite in rat plasma. J. Chromatogr. B Analyt. Technol. Biomed. Life Sci..

[58] Hajishengallis G. (2020). New developments in neutrophil biology and periodontitis. Periodontol. 2000.

[59] Pohl P., Joshi R., Petrvalska O., Obsil T., Obsilova V. (2021). 14-3-3-Protein regulates Nedd4-2 by modulating interactions between HECT and WW domains. Commun. Biol..

[60] Bhalla V., Daidie D., Li H., Pao A.C., LaGrange L.P., Wang J. (2005). Serum- and glucocorticoid-regulated kinase 1 regulates ubiquitin ligase neural precursor cell-expressed, developmentally down-regulated protein 4-2 by inducing interaction with 14-3-3. Mol. Endocrinol..

[61] Fajner V., Maspero E., Polo S. (2017). Targeting HECT-type E3 ligases - insights from catalysis, regulation and inhibitors. FEBS Lett..

[62] Xia Z.P., Chen Z.J. (2005). TRAF2: a double-edged sword?. Sci. STKE.

[63] Li J., Ayoub A., Xiu Y., Yin X., Sanders J.O., Mesfin A. (2019). TGFbeta-induced degradation of TRAF3 in mesenchymal progenitor cells causes age-related osteoporosis. Nat. Commun..

[64] Parvatiyar K., Pindado J., Dev A., Aliyari S.R., Zaver S.A., Gerami H. (2018). A TRAF3-NIK module differentially regulates DNA vs RNA pathways in innate immune signaling. Nat. Commun..

[65] Hacker H., Redecke V., Blagoev B., Kratchmarova I., Hsu L.C., Wang G.G. (2006). Specificity in Toll-like receptor signalling through distinct effector functions of TRAF3 and TRAF6. Nature.

[66] Oganesyan G., Saha S.K., Guo B., He J.Q., Shahangian A., Zarnegar B. (2006). Critical role of TRAF3 in the Toll-like receptor-dependent and -independent antiviral response. Nature.

[67] Li C., Yang X., Pan Y., Yu N., Xu X., Tong T. (2017). A sialidase-deficient Porphyromonas gingivalis mutant strain induces less interleukin-1beta and tumor necrosis factor-alpha in Epi4 cells than W83 strain through regulation of c-Jun N-terminal kinase pathway. J. Periodontol..

[68] Okahashi N., Inaba H., Nakagawa I., Yamamura T., Kuboniwa M., Nakayama K. (2004). Porphyromonas gingivalis induces receptor activator of NF-kappaB ligand expression in osteoblasts through the activator protein 1 pathway. Infect. Immun..

[69] Tessier M., Woodgett J.R. (2006). Serum and glucocorticoid-regulated protein kinases: variations on a theme. J. Cell. Biochem..

[70] Wang H., Garcia C.A., Rehani K., Cekic C., Alard P., Kinane D.F. (2008). IFN-beta production by TLR4-stimulated innate immune cells is negatively regulated by GSK3-beta. J. Immunol..

[71] Liu S., Chen Z.J. (2011). Expanding role of ubiquitination in NF-kappaB signaling. Cell Res..

[72] Song H.Y., Rothe M., Goeddel D.V. (1996). The tumor necrosis factor-inducible zinc finger protein A20 interacts with TRAF1/TRAF2 and inhibits NF-kappaB activation. Proc. Natl. Acad. Sci. U. S. A..

[73] Kany S., Vollrath J.T., Relja B. (2019). Cytokines in inflammatory disease. Int. J. Mol. Sci..

[74] Ackermann T.F., Boini K.M., Beier N., Scholz W., Fuchss T., Lang F. (2011). EMD638683, a novel SGK inhibitor with antihypertensive potency. Cell. Physiol. Biochem..

[75] Weischenfeldt J., Porse B. (2008). Bone marrow-derived macrophages (BMM): isolation and applications. CSH Protoc..

[76] Kirkby N.S., Chan M.V., Zaiss A.K., Garcia-Vaz E., Jiao J., Berglund L.M. (2016). Systematic study of constitutive cyclooxygenase-2 expression: role of NF-kappaB and NFAT transcriptional pathways. Proc. Natl. Acad. Sci. U. S. A..

